# Daily Oral Administration of Protease-Treated Royal Jelly Protects Against Denervation-Induced Skeletal Muscle Atrophy

**DOI:** 10.3390/nu12103089

**Published:** 2020-10-11

**Authors:** Tomohiko Shirakawa, Aki Miyawaki, Takuma Matsubara, Nobuaki Okumura, Hideto Okamoto, Naoya Nakai, Thira Rojasawasthien, Kazumasa Morikawa, Asako Inoue, Akino Goto, Ayako Washio, Toshiyuki Tsujisawa, Tatsuo Kawamoto, Shoichiro Kokabu

**Affiliations:** 1 Division of Molecular Signaling and Biochemistry, Department of Health Improvement, Kyushu Dental University, Kitakyushu, Fukuoka 803-8580, Japan; r16shirakawa@fa.kyu-dent.ac.jp (T.S.); r17miyawaki@fa.kyu-dent.ac.jp (A.M.); r15matsubara@fa.kyu-dent.ac.jp (T.M.); s17nakai@st.kyu-dent.ac.jp (N.N.); r19Rojasawasthien@fa.kyu-dent.ac.jp (T.R.); r14inoue2@fa.kyu-dent.ac.jp (A.I.); r18goto@fa.kyu-dent.ac.jp (A.G.); 2 Division of Orofacial Functions and Orthodontics, Department of Health Improvement, Kyushu Dental University, Kitakyushu, Fukuoka 803-8580, Japan; r15kawamoto@fa.kyu-dent.ac.jp; 3 Institute for Bee Products and Health Science, Yamada Bee Company, Inc., Tomata, Okayama 708-0393, Japan; no1780@yamada-bee.com (N.O.); ho1993@yamada-bee.com (H.O.); 4 Division of Pediatric and Special Care Dentistry, Department of Developmental Oral Health Science, School of Dentistry, Iwate Medical University, Shiwa, Iwate 028-3694, Japan; kazumasa@iwate-med.ac.jp; 5 Division of Endodontics and Restorative Dentistry, Department of Oral Functions, Kyushu Dental University, Kitakyushu, Fukuoka 803-8580, Japan; r05washio@fa.kyu-dent.ac.jp; 6 School of Oral Health Sciences, Kyushu Dental University, Kitakyushu, Fukuoka 803-8580, Japan; t-toshi@kyu-dent.ac.jp

**Keywords:** skeletal muscle, royal jelly, myoblasts, atrophy, denervation

## Abstract

Honeybees produce royal jelly (RJ) from their cephalic glands. Royal jelly is a source of nutrition for the queen honey bee throughout its lifespan and is also involved in fertility and longevity. Royal jelly has long been considered beneficial to human health. We recently observed that RJ delayed impairment of motor function during aging, affecting muscle fiber size. However, how RJ affects skeletal muscle metabolism and the functional component of RJ is as of yet unidentified. We demonstrate that feeding mice with RJ daily prevents a decrease in myofiber size following denervation without affecting total muscle weight. RJ did not affect atrophy-related genes but stimulated the expression of myogenesis-related genes, including *IGF-1* and *IGF receptor*. Trans-10-hydroxy-2-decenoic acid (10H2DA) and 10-hydroxydecanoic acid (10HDAA), two major fatty acids contained in RJ. After ingestion, 10H2DA and 10HDAA are metabolized into 2-decenedioic acid (2DA) and sebacic acid (SA) respectively. We found that 10H2DA, 10HDAA, 2DA, and SA all regulated myogenesis of C2C12 cells, murine myoblast cells. These novel findings may be useful for potential preventative and therapeutic applications for muscle atrophy disease included in Sarcopenia, an age-related decline in skeletal muscle mass and strength.

## 1. Introduction

Sarcopenia is an age-related decline in skeletal muscle mass and strength [1]. Loss of muscle mass gives rise to adverse consequences such as increased insulin resistance, poor quality of life, dependency, hospitalization, and ultimately an increase in mortality [2]. Muscle fiber degeneration and impaired satellite cell regeneration contribute to Sarcopenia. Muscle fiber degeneration is mostly a consequence of neuromuscular dysfunction and denervation [3,4,5], while impaired satellite-cell regenerative capacity is due to a combination of reduced satellite cell numbers and decreased differentiation potential [6,7,8]. With the rapid aging of society worldwide, there is an urgent need for therapeutic strategies that will improve skeletal muscle mass and function in aging adults. 

Satellite cells are skeletal muscle stem cells that reside beneath the basal lamina. Satellite cells play a central role in postnatal muscle growth, repair, and regeneration in adults. Upon activation, satellite cells proliferate extensively and upregulate expression of MyoD, followed by increasing myogenic differentiation marker genes such as myogenin, muscle creatine kinase (Mck), and myosin heavy chain (Myhc) [9,10,11]. Under conditions of skeletal muscle atrophy, such as Sarcopenia, the rate of muscle fiber loss or degradation surpasses that of de novo myogenesis of satellite cells. Thus, the attenuation of catabolic process and/or stimulation of anabolic process in skeletal muscle metabolism are potential candidates for the treatment for skeletal muscle atrophy diseases. 

Honeybees (e.g., *Apis mellifera*) produce royal jelly (RJ) from their cephalic glands. Royal jelly is a source of nutrition for the queen honey bee throughout its lifespan and is also involved in fertility and longevity. RJ has long been considered beneficial to health [12,13]. Animal experiments suggest that RJ prolongs life span [14,15], reduces fatigue [16], and contains antioxidant and anti-inflammatory properties [17,18,19]. In human trials, RJ decreases total serum cholesterol and total serum lipids [20]. 

Royal jelly is composed of water (60–70%), proteins (9–18%), sugars (7.5–23%), lipids (3–8%), and other trace compounds. The two major fatty acids in RJ are trans-10-hydroxy-2-decenoic acid (10H2DA) and 10-hydroxydecanoic acid (10HDAA), which comprise 60–80% of RJ lipids [21]. 10H2DA and 10HDAA have been shown to be pharmacologically active in animal experiments, thus providing a possible mechanism for the therapeutic effects of RJ [22,23,24,25,26]. In contrast, proteins contained in RJ occasionally induce anaphylactic reaction [27,28,29]. Major royal jelly protein 1(MRJP) is a frequent allergen for honey-related allergies [30]. To eliminate such adverse events with RJ supplementation, protease-treated RJ (pRJ) has been developed by treating RJ with alkaline proteases, leading to complete elimination of MRJP without nutritional loss of minerals, vitamins, and fatty acids [31].

Royal jelly also appears to have a function in skeletal muscle metabolism. In mice, feeding of RJ increases the serum IGF1 levels and stimulates regeneration of injured muscle via the IGF1-Akt pathway in satellite cells [32]. Administration of RJ also induces mitochondrial adaptation with endurance training by adenosine monophosphate-activated protein kinase (AMPK) activation in the soleus muscle of ICR mice [33]. Human clinical trials demonstrated that RJ has the potential to attenuate the age-related decline in grip strength [34]. We recently compared the effects of enzyme-untreated RJ (NRJ) with pRJ on motor functions of aging mice and observed that both NRJ and pRJ delayed impairment of motor function during aging [35]. Furthermore, RJ treatment affected muscle fiber size as well as the expression of satellite cell markers and catabolic genes [35]. 

Here, we demonstrate that daily feeding of pRJ in mice cancels the in of muscle fiber size induced by denervation. In addition, treatment of C2C12 myoblasts with pRJ and pRJ-related fatty acids stimulated differentiation and proliferation. 

## 2. Material and Methods

### 2.1. Denervation Model

C57BL/6J mice were purchased from CLEA Japan Inc. (Tokyo, Japan). Seven-week-old mice were anesthetized, after which a 5 mm section of the sciatic nerve on the right leg was cut and excised. A sham operation was performed on the left leg as a control [36]. Six days later, muscles were removed and immediately frozen in isopentane cooled in liquid nitrogen or prepared for RNA extraction. All mice were used in accordance with guidelines from the Kyushu Dental University Animal Care and Use Committee. All experiments were carried out with the approval of the Animal Use and Care Committee of the Kyushu Dental University (Approval number #18–33).

### 2.2. Experimental Diet and pRJ Treatment 

Lyophilized protease-treated RJ (pRJ, Lot No. YDP-M-170610) was prepared at Yamada Bee Company, Inc. (Okayama, Japan). Protease-treated RJ contained a standardized amount of specific fatty acids (3.5% 10H2DA and 0.6% 10HDAA). Experimental diets were prepared by thoroughly mixing pRJ with MF powder diet (Oriental Yeast, Tokyo, Japan) at a concentration of 1% (v/v) [35]. Four-week-old C57BL/6 mice were fed control diet (*n* = 7) or 1% pRJ diet (*n* = 7) for 3 weeks pre-operation and for 6 days post-operation. Chow was refreshed every 2 days. 

### 2.3. Histochemical Analysis

Tibialis anterior (TA) muscle was isolated after sacrifice and immediately frozen in chilled isopentane and liquid nitrogen and stored at −80 °C [11]. Sections were stained with hematoxylin and eosin (H&E). Images of sections were digitally captured with a BZ-II Analyzer (KEYENCE, Osaka, Japan). The circumference of each fiber was outlined using ImageJ software (National Institute for Health) to generate cross sectional area (CSA) of myofibers. Criteria for the selection of muscle fibers to determine for CSA of myofibers included an intact, distinct cell membrane without significant signs of folding or distortion. Elongated fibers indicating an oblique section were also excluded. Image analysis was performed by two authors (A. M. and T. S.).

### 2.4. Cell Culture, Reagents, and Skeletal Muscle Differentiation

C2C12 cells and C3H10T1/2 cells were purchased from American Type Culture Collection (Manassas, VA, USA). C2C12 cells and C3H10T1/2 cells were maintained as previously described [11] and cultured in the presence of 0, 0.25, 0.5, or 1.0 mg/ml pRJ solution. Fatty acids, where indicated, were used at 500 μM [37]. pRJ (Lot No. YDP-M-170610), Trans-10-hydroxy-2-decenoic acid (10H2DA), 10-hydroxydecanoic acid (10HDAA), 2-decenedioic acid (2DA), and sebacic acid (SA), were prepared at Yamada Bee Company, Inc. (Okayama, Japan). Decanoic acid (DA) and docosahexaenoic acid (DHA) were obtained from Fujifilm wako chemicals (Osaka, Japan). 

Skeletal muscle differentiation in C2C12 cells was initiated by replacing growth medium (medium supplemented with 10% fetal bovine serum) with differentiation medium (medium supplemented with 2% horse serum) in sub-confluent cultures [11]. 

### 2.5. RNA Isolation and Quantitative Real-Time PCR (qPCR)

Total RNA was isolated from cells using a FastGeneTM RNA Basic Kit (Nippon Genetics, Tokyo, Japan) and then reverse-transcribed into cDNA using High Capacity cDNA Reverse Transcription Kit (Applied biosystems). SYBR green-based qPCR was performed in 96-well plates using PowerUp SYBR Green Master Mix (ThermoFisher Scientific, Waltham, MA, USA) and a QuantStudio 3 Real-Time PCR System (ThermoFisher Scientific). Expression levels were normalized to *TATA box binding protein* (*Tbp*) using the 2^-^*^ΔΔ^*^Ct^ method [38]. The following primers were used for qPCR analyses: *murine atrogin-1* (primer sequences: forward, agtgaggaccggctactgtg; reverse, gatcaaacgcttgcgaatct), *murine murf1* (primer sequences: forward, tgacatctacaagcaggagtgc; reverse, tcgtcttcgtgttccttgc), *murine foxo1* (primer sequences: forward, cttcaaggataagggcgaca; reverse, gacagattgtggcgaattga), *murine myogenin* (primer sequences: forward, ccttgctcagctccctca; reverse, tgggagttgcattcactgg), *murine myoD* (primer sequences: forward, agcactacagtggcgactca; reverse, ggccgctgtaatccatcat), *murine mck* (primer sequences: forward, cagcacagacagacactcagg; reverse, gaacttgttgtgggtgttgc), *murine* *cyclin A2* (primer sequences: forward, cttggctgcaccaacagtaa; reverse, caaactcagttctcccaaaaaca), *murine cyclin D1* (primer sequences: forward, tttctttccagagtcatcaagtgt; reverse, tgactccagaagggcttcaa), *murine cyclin E1* (primer sequences: forward, tttctgcagcgtcatcctc; reverse, tggagcttatagacttcgcaca), *murine Myhc1* (primer sequences: forward, aatcaaaggtcaaggcctacaa; reverse, gaatttggccaggttgacat), *murine Myhc7* (primer sequences: forward, cgcatcaaggagctcacc; reverse, ctgcagccgcagtaggtt), *murine Myhc2* (primer sequences: forward, aactccaggcaaaagtgaaatc; reverse, cttggatagatttgtgttggattg), *murine Myhc4* (primer sequences: forward, aacccttaaagtacttgtctgactcaa; reverse, gctattggtggcagctcag), *murine IGF1* (primer sequences: forward, agcagccttccaactcaattat; reverse, tgaagacgacatgatgtgtatctttat), *murine IGF1R* (primer sequences: forward, gagaatttccttcacaattccatc; reverse, cacttgcatgacgtctctcc), and *murine tbp* (primer sequences: forward, ggcggtttggctaggttt; reverse, gggttatcttcacacaccatga).

### 2.6. Immunocytochemistry Analysis

C2C12 cells were incubated with primary antibody for 1 hour at room temperature after blocking and permeabilization with phosphate-buffered saline containing 0.3% Triton X100 and 5% goat serum for 30 minutes at room temperature. Anti-Myhc mouse monoclonal antibody (MF20, R & D Systems, Minneapolis, MN, USA) or anti-Ki-67 rabbit polyclonal antibody (ab15580, Abcam) were used for immunocytochemistry. Target proteins were visualized using Alexa 488-conjugated secondary antibody (Invitrogen, Carlsbad, CA, USA) and imaged with an ABZ-9000 (Keyence, Tokyo, Japan) microscope.

### 2.7. Western Blot Analysis 

Antibodies used for Western blot analysis were anti-Myogenin mouse monoclonal antibody (F5D, Santa Cruz, Santa Cruz, CA, USA), anti-Myhc mouse monoclonal antibody (MF20, R & D systems, Minneapolis, MN), anti-CyclinD1 mouse monoclonal antibody (72-13G, Santa Cruz), anti-Cyclin A2 rabbit polyclonal antibody (GST103042, GenTex, Irvine, CA, USA), Phosopho-anti-AMPKα (Thr172) Rabbit monoclonal antibody (40H9, Cell Signaling), anti-AMPKα Rabbit monoclonal antibody (D63G4, Cell Signaling), anti-Fbx32 (Atrogin-1) Rabbit monoclonal antibody (ab168372, Abcam), and HRP-conjugated anti-Gapdh mouse monoclonal antibody (Proteintech, Chicago, IL, USA). Target proteins were detected using anti-mouse or anti-rabbit IgG antibody conjugated with horseradish peroxidase (Cell signaling, Beverly, MA, USA) and ImmunoStar LD (Fujifilm wako chemicals, Osaka, Japan).

### 2.8. Cell Proliferation Assay

Proliferation of C2C12 cells was assessed using a Cell Counting kit-8 (Dojindo, Kumamoto, Japan) according to the manufacturer’s protocol [39]. 

### 2.9. Statistical Analysis

Comparisons were made using an unpaired analysis of variance (ANOVA) with Tukey–Kramer post-hoc test and Wilcoxon’s signed rank test. The results are shown as the mean ± S.D. The statistical significance is indicated as follows: **, *p* < 0.01 and *, *p* < 0.05.

## 3. Results

### 3.1. pRJ Attenuates Denervation-Induced Skeletal Muscle Atrophy

**Figure 1:**
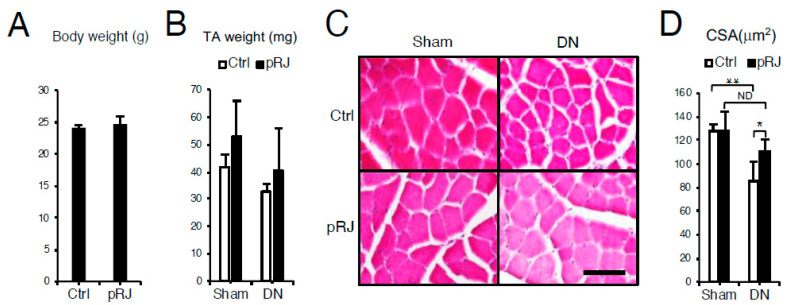
Protease-treated royal jelly (pRJ) prevents skeletal muscle atrophy following denervation. The effect of dietary protease-treated royal jelly (pRJ) on (**A**) total body weight and (**B**) wet weight of tibialis anterior (TA) muscles following denervation (DN). (**C**) Representative images of TA muscle cross-sections and (**D**) quantification of muscle cross-sectional area (CSA). Scale = 100 μm. Data are mean ± SD (*n* = 10). ∗∗*p* < 0.01, versus sham-operated (Sham). ∗*p* < 0.05, versus control (Ctrl). No significant difference (ND), versus Sham.

**Figure 2:**
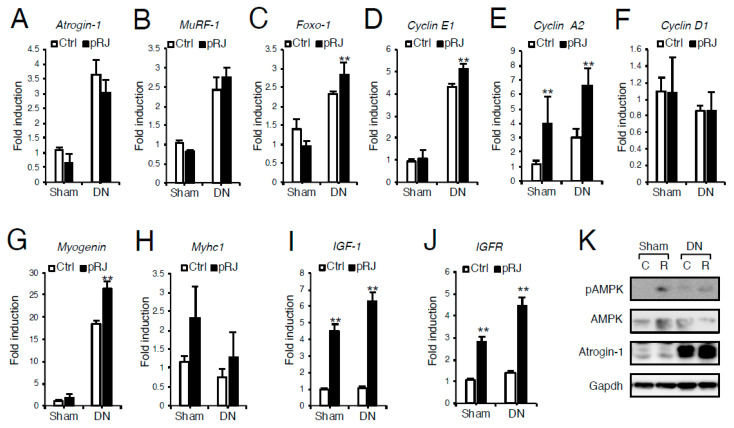
pRJ increased regeneration gene expression but does not influence atrophy gene expression. (**A**–**J**) qPCR analysis of mRNA levels of *Atrogin-1* (**A**), *Murf1* (**B**), *Foxo-1* (**C**), *Cyclin E1*(**D**), *Cyclin A2* (**E**), *Cyclin D1* (**F**), *Myogenin* (**G**), *Myhc1*(**H**), *IGF-1*(**I**), or *IGF receptor (IGFR)*(**J**) in sham or denervated muscle with or without 1% pRJ feeding. Western blotting analysis showed the protein levels of pAMPK, AMPK, Atrogin-1, and Gapdh in sham or denervated muscle with or without 1% pRJ feeding (**K**). Data are mean ± SD (*n* = 7). ∗∗*p* < 0.01, ∗*p* < 0.05, versus control (Ctrl).

To examine the effect of pRJ on skeletal muscle atrophy, C57BL/6 mice were fed on control or pRJ diets for four weeks, and then muscle atrophy was induced by denervation. Daily feeding of pRJ for 1 month had no significant effect on total body weight (Figure 1A) or loss of total tibialis anterior muscle weight induced by denervation (Figure 1B). However, pRJ prevented the decrease in skeletal muscle fiber diameter following denervation (Figure 1C,D). In order to determine the mechanism by which pRJ prevents the decrease in muscle fiber size, we next compared the expression levels of atrophy, proliferation, or skeletal muscle differentiation genes. qPCR and Western blotting analysis showed that pRJ did not alter the expression of catabolic genes such as *Atrogin-1* (Figure 2A and 2K), *Muscle ring finger protein 1* (*MuRF1*) (Figure 2B), or *Forkhead box O-1* (*Foxo-1*) (Figure 2C). However, pRJ increased the expression of proliferation and differentiation-related genes such as *Cyclin E1* (Figure 2D), *Cyclin A2* (Figure 2E), or *Myogenin* (Figure 2G). Protease-treated RJ had no significant effect on the upregulation of *Mych1* (Figure 2H), *Myhc2* (Supplementary Figure S1A), *Myhc4* (Supplementary Figure S1B), or *Myhc7* (Supplementary Figure S1C). Protease-treated RJ stimulated the upregulation of *IGF-1* (Figure 2I) and *IGF receptor* (*IGFR*) (Figure 2J) and phosphorylation of AMPK (Figure 2K). 

### 3.2. pRJ Stimulates Myoblast Proliferation

Next, we examined the effect of pRJ on proliferation using an in vitro cell culture system. C2C12 cells are from a murine myoblast cell line isolated from satellite cells [40] commonly used as an in vitro model of muscle regeneration. Proliferating C2C12 cells will cease proliferation and promptly differentiate into myofibers upon stimulation in a manner similar to satellite cells [11]. 

**Figure 3:**
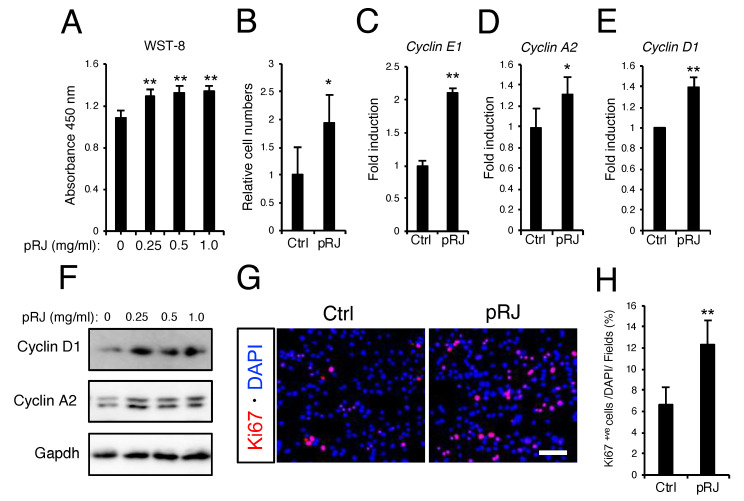
pRJ stimulates proliferation of myoblasts in C2C12 cells. (**A**–**B**) C2C12 cells were treated with 0. 0.25, 0.5, or 1.0 mg/mL pRJ solution for 2 days. The number of living cells was assessed using Cell Counting kit-8. Cells were treated with 1.0 mg/mL pRJ for 2 days. After staining with trypan blue, the number of living cells was determined by direct counting. Graphs show the ratio of number of cells treated with pRJ divided by control (**B**). (**C**–**E**) The mRNA levels of indicated genes in cells treated with or without 1.0 mg/ml pRJ for 2 days. (**F**) Western blot showing protein levels of Cyclin D1, Cyclin A2, or Gapdh in C2C12 cells treated with 0. 0.25, 0.5, or 1.0 mg/mL pRJ solution for 2 days. (**G** and **H**) Images of Ki67 positive (+ve) immunostaining in cells treated with or without 1.0 mg/mL pRJ (**G**). The graph indicates the number of Ki67^+ve^ cells as a percentage of total cells stained with DAPI (**H**). Images are representative of multiple independent experiments (**F** and **G**). Scale bar corresponds to 100 μm (**G**). Data are mean ± SD (*n* = 4). ∗∗*p* < 0.01, ∗*p* < 0.05, versus control (Ctrl).

**Figure 4:**
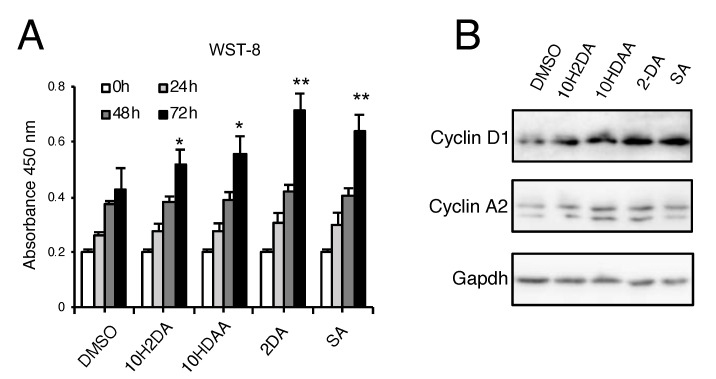
RJ-related fatty acids stimulate proliferation in C2C12 cells. (**A**) C2C12 cells were treated with 500 μM 10H2DA, 10HDAA, 2DA, or SA for 0, 24, 48, or 72 hours after which the number of living cells was assessed using a Cell-Counting kit-8. (**B**) Cells were treated with indicated fatty acids for 2 days, and the protein levels of Cyclin D1, Cyclin A2, and Gapdh were determined by Western blotting (**B**). Data are mean ± SD (*n* = 4). ∗∗*p* < 0.01, ∗*p* < 0.05, versus Dimethyl sulfoxide (DMSO) treatment (**A**). Images are representative of multiple independent experiments (**B**). Abbreviations: 10H2DA: Trans-10-hydroxy-2-decenoic acid; 10HDAA: 10-hydroxydecanoic acid; 2DA: 2-decenedioic acid; SA: Sebacic acid.

Protease-treated royal jelly treatment for 48 hours increased the number of cells (Figure 3A,B) as well as the expression of cell-cycle-related genes (Figure 3C–F). Furthermore, Ki67 immunostaining showed that pRJ treatment increased the number of Ki67-positive proliferative cells (Figure 3G,H). Trans-10-hydroxy-2-decenoic acid and 10-hydroxydecanoic acid are two fatty acids specifically occurring in RJ. Trans-10-hydroxy-2-decenoic acid and 10-hydroxydecanoic acid can be metabolized into 2DA and SA, respectively [41]. To determine whether RJ-derived fatty acids and/or their metabolic products affect proliferation, C2C12 cells were treated with the indicated compounds for 24, 48, and 72 hours, after which cell numbers were quantified by WST-8. As shown in Figure 4A, 10H2DA, 10HDAA, 2DA, and SA all significantly increased cell proliferation (Figure 4A). These RJ-related fatty acids also increased the protein levels of cell cycle genes such as Cyclin D1 and Cyclin A2 (Figure 4B).

### 3.3. pRJ Stimulates Myoblast Differentiation

**Figure 5:**
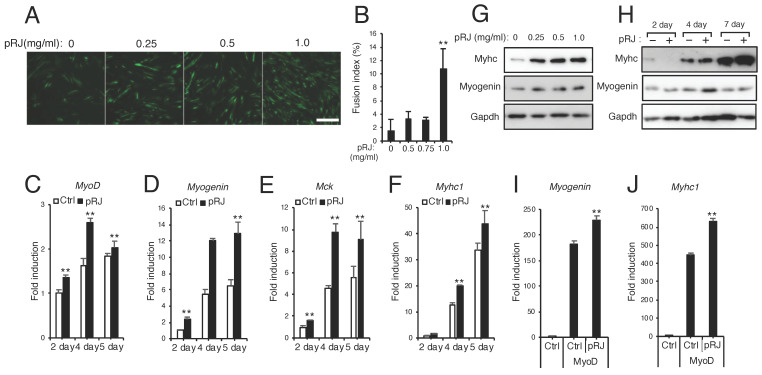
pRJ stimulates myoblast differentiation. (**A**,**B**) C2C12 cells were treated with myogenic medium supplemented with pRJ at 0, 0.25, 0.5, or 1.0 mg/mL for 6 days. Cells were then stained with anti-myosin heavy chain antibody (**A**). Fusion index was quantified as the number of nuclei (at least three) within myotubes divided by the total number of nuclei (**B**). (**C**–**F**) Cells were treated with myogenic medium together with or without 1 mg/ml pRJ for 2, 4, or 5 days. mRNA levels of the indicated myogenic markers were determined by qPCR. (**G** and **H**) C2C12 cells were treated with myogenic medium supplemented with pRJ at 0, 0.25, 0.5, or 1.0 mg/mL for 4 days (**G**) or treated with 1 mg/mL pRJ for 2, 4, or 7 days (**H**). Western blots showing protein levels of myosin heavy chain (Myhc), Myogenin, or Gapdh (**G** and **H**). (**I** and **J**) C3H10T1/2 cells were transfected with or without MyoD and then treated with or without 1mg/mL pRJ. mRNA levels of *Myogenin* or *Myhc1* were determined on day 2 by qPCR. Representative images are shown. Scale = 100 μm (**A**, **G**, and **H**). Data are mean ± SD (*n* = 4). ∗∗*p* < 0.01, ∗*p* < 0.05, versus control (Ctrl).

**Figure 6:**
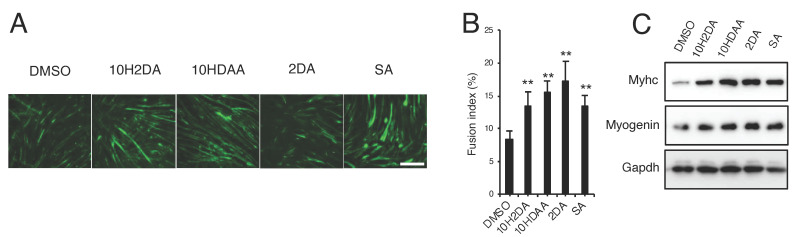
RJ-related fatty acids increase myoblast differentiation in C2C12 cells. (**A**–**C**) C2C12 cells were treated with myogenic medium along with 500 μM 10H2DA, 10HDAA, 2DA, or SA for 6 days. Cells were then stained with anti-myosin heavy chain antibody (**A**). Fusion index was quantified as the number of nuclei within myotubes (at least three) divided by the total number of nuclei (B). Western blotting showing protein levels of myosin heavy chain (Myhc), Myogenin, or Gapdh determined (**C**). Representative images are shown. Scale = 100 μm (**A** and **C**). Data are mean ± SD (*n* = 4). ∗∗*p* < 0.01, versus DMSO treatment.

Finally, we examined the effect of pRJ and RJ-related fatty acid products on myoblast differentiation. C2C12 cells were induced to differentiate in the presence or absence of pRJ. Myosin heavy-chain immunostaining showed that pRJ treatment led to an increase in myotube formation compared to control treatment cells (Figure 5A,B). Furthermore, pRJ treatment elevated the expression level of muscle differentiation genes such as MyoD, Myogenin, Mck, or Myhc1 (Figure 5C–H). C3H10T1/2 cell model is a mouse embryonic fibroblast cell line with myogenic potential. C3H10T1/2 cells, however, do not normally express MyoD, a master regulator of myogenesis [42]. To evaluate the effect of pRJ on MyoD function, we overexpressed MyoD in C3H10T1/2 cells and then treated the cells with or without pRJ. qPCR analysis revealed that pRJ treatment enhanced the induction of Myogenin and Myhc1 induced by MyoD (Figure 5I,J). Treatment with 10H2DA, 10HDAA, 2DA, and SA increased myotube formation (Figure 6A,B). The treatment of cells with these RJ-related fatty acids increased the protein levels of myogenic differentiation marker genes such as Myhc and myogenin (Figure 6C). 

## 4. Discussion

In this study, we demonstrate that feeding mice daily with pRJ prevents a decrease in myofiber size following denervation. In a previous study, we showed that pRJ affects muscle fiber size in elderly mice without changing total muscle weight [35]. Our current findings did not conflict with this study. Muscle weight during pathological conditions such as obesity, type 2 diabetes, or age-related Sarcopenia can be affected by the infiltration of adipose and/or connective tissue [43]. In our study, we did not quantify the infiltration of adipose or connective tissue into the muscle tissue, even though pRJ treatment tended to increase muscle weight. It may be interesting to explore the effect of RJ on the infiltration of muscle by adipose and connective tissue. Our data showed that daily feeding with pRJ did not decrease the expression levels of atrophy-related genes such as Atrogin-1, Murf1, or Foxo-1 although our previous work showed that pRJ feeding decreases atrophy-related gene expression during aging [35]. This discrepancy might be explained by differences in the relative extent of atrophy induced by denervation in comparison to aging. Nevertheless, differences between the two experimental models may be helpful to clarify the effect of RJ on skeletal muscle metabolism.

10-hydroxy-2-decenoic acid and 10-hydroxydecanoic acid, two major fatty acids contained in RJ, are associated with health benefits such as anti-tumor activity [22], anti-hypersteatosis activity [23], antibiotic activity [25], and anti-depression activity [26] in vitro and in vivo. After ingestion, 10H2DA and 10HDAA are metabolized into 2DA and SA, respectively. 2-decenedioic acid and sebacic acid can be detected in human plasma and urine samples, but 10H2DA and 10HDAA are not detected following RJ intake [41]. Therefore, in cell culture models, 2DA and SA are useful in exploring the function of 10H2DA and 10HDAA. In our present study, we found that 10H2DA, 10HDAA, 2DA, and SA all regulated myogenesis of C2C12 cells, suggesting that fatty acids from RJ may have a stronger effect on myogenesis than others. Interestingly, decanoic acid (DA), which is non-hydroxylated at the C-terminal, unlike RJ-derived decanoic acid (10HDAA), did not affect differentiation of C2C12 cells at equimolar concentrations as RJ derived fatty acids (Supplementary Figure S2). These novel findings may be useful for potential preventative and therapeutic applications for muscle atrophy since these RJ fatty acids stimulate proliferation and differentiation myoblasts. 

We observed that RJ treatment stimulated both proliferation and differentiation. RJ stimulates cell proliferation and increases the size of Myhc positive fibers in primary satellite cells isolated from aged mice via upregulation of IGF-1 and IGFR [32]. In this study, RJ treatment significantly increased expression of IGF-1 and IGFR, suggesting that the IGF1-Akt pathway may contribute to the phenotype. In in vivo experiments, RJ treatment did not increase the expression levels of cyclin D1, whereas RJ treatment in vitro strongly increased cyclinD1. Currently, it is difficult to explain this discrepancy. Furthermore, we could not reveal which cell types are proliferating and differentiating in vivo following RJ treatment. This will be an important issue to resolve in future studies.

Royal jelly has been known to regulate global epigenetic changes [44]. Epigenetic status is maintained by the enzymes such as DNA methyltransferases, histone acetylases and deacetylases, and histone methyltransferases and demethylases. These enzymes can be targeted by nutritional factors [45]. Royal jelly and 10H2DA can inhibit histone deacetylase (HDAC) activity without affecting DNA methylation [46]. As described above, 10H2DA possesses anti-tumor activity [22]. Interestingly, valproic acid, an HDAC inhibitor, also inhibits angiogenesis in malignant tumors [47], and several HDAC inhibitors are used in cancer treatment [48]. Inhibition of DNA methyltransferases and HDAC have positive effects on myogenesis [49,50,51,52,53]. In our preliminary data, RJ treatment enhanced myoblast differentiation in C2C12 cells synergistically with 5-aza-2-deoxycytidine, a DNA methyltransferase inhibitor (data not shown). However, RJ treatment could not increase differentiation in the presence of Trichostatin A, an HDAC inhibitor (data not shown), suggesting that RJ may stimulate myoblast differentiation via regulating HDAC activity. Further experiments are required to elucidate the mechanism by which RJ regulates skeletal muscle metabolism. 

In conclusion, daily oral administration of pRJ prevented a decrease in myofiber size following denervation. pRJ also increased the expression of regeneration-related genes in vivo. Although we could not determine the proliferative cell population responding to pRJ in vivo, pRJ and RJ related fatty acids strongly stimulated proliferation and differentiation of C2C12 myoblasts in vitro.

## Supplementary Materials

The following are available online at https://www.mdpi.com/2072-6643/12/10/3089/s1, Figure S1: mRNA levels of myosin heavy chains in vivo. Figure S2: Decanoic acid promotes the proliferation in C2C12 cells.

## Abbreviations

Royal jelly	RJ
Protease-treated RJ	pRJ
Trans-10-hydroxy-2-decenoic acid	10H2DA
10-hydroxydecanoic acid	10HDAA
2-decenedioic acid	2DA
Sebacic acid	SA
Decanoic acid	DA
Docosahexaenoic acid	DHA
Muscle creatine kinase	Mck
Myosin heavy chain	Myhc
Muscle ring finger protein 1	Murf1
Forkhead box O1	Foxo1
Quantitative real time PCR	qPCR
messenger RNA	mRNA
Insulin-like growth factor-1	IGF-1
Insulin-like growth factor receptor	IGFR
