# Supplementary material for: Daily Oral Administration of Protease-Treated Royal Jelly Protects Against Denervation-Induced Skeletal Muscle Atrophy
Source: Nutrients. 2020 Oct 11;12(10):3089. doi: 10.3390/nu12103089 (PMC7600733; doi:10.3390/nu12103089)
Supplement: Supplementary file 1 [file nutrients-12-03089-s001.pdf]

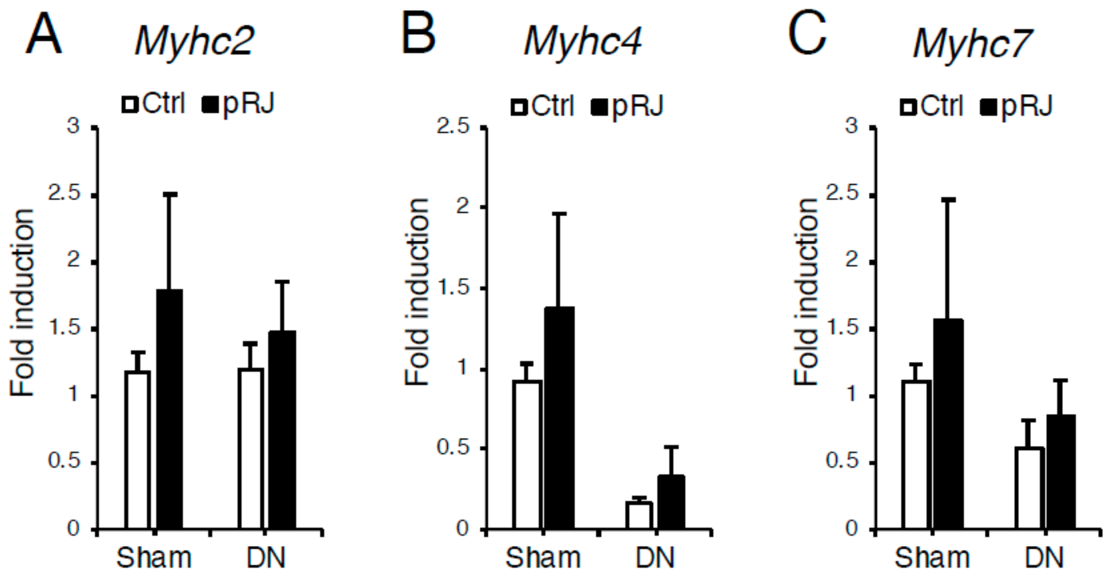

Supplemental Figure S1. mRNA levels of myosin heavy chains *in vivo*. (A - C) qPCR analysis of mRNA levels of *myosin heavy chain 2 (Myhc2)* (A), *myosin heavy chain 4 (Myhc4)* (B), *myosin heavy chain 7 (myhc7)* (C) in sham or denervated muscle with or without 1% pRJ. Data are mean ± SD (n = 7).

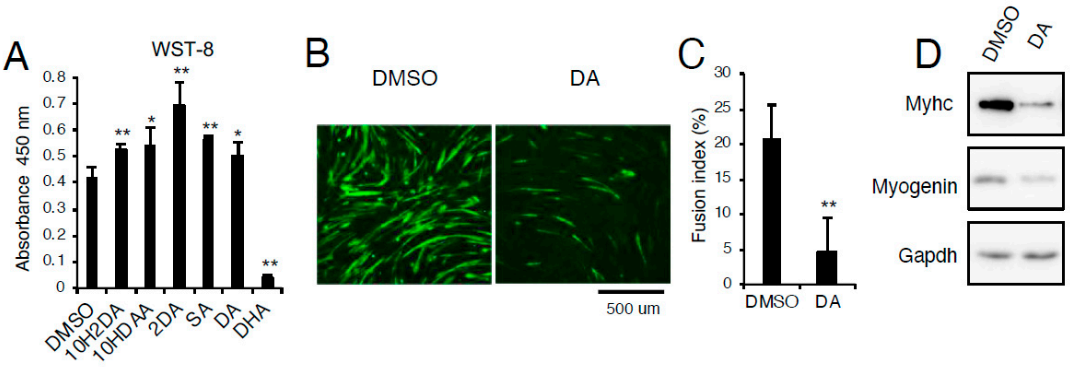

Supplemental Figure S2. Decanoic acid promotes the proliferation in C2C12 cells

(A) C2C12 cells were treated with 500 μM 10H2DA, 10HDAA, 2DA, SA, decanoic acid (DA) or docosahexaenoic acid (DHA) for 72 hours after which the number of living cells were assessed using a Cell Counting kit-8. (B - D) Cells were treated with myogenic medium supplemented with or without 500 μM DA for 6 days. Cells were then stained with anti-myosin heavy chain antibody (B). Fusion index were quantified as the number of nuclei within myotubes divided by the total number of nuclei (C). Western blotting showing protein levels of myosin heavy chain (Myhc), Myogenin, or Gapdh determined (D). Representative images are shown. Scale = 100 μm (B and D). Data are mean ± SD (n = 4). $**P < 0.01$, $*P < 0.05$, versus DMSO treatment.
